# Anticholinergic Drugs Interact With Neuroprotective Chaperone L-PGDS and Modulate Cytotoxicity of Aβ Amyloids

**DOI:** 10.3389/fphar.2020.00862

**Published:** 2020-06-11

**Authors:** Kimberly Jia Yi Low, Margaret Phillips, Konstantin Pervushin

**Affiliations:** School of Biological Sciences, Nanyang Technological University, Singapore, Singapore

**Keywords:** anticholinergic, anticholinergic adverse effect, amyloid, lipocalin-type prostaglandin D synthase, Alzheimer’s disease, amyloid beta (Aβ)

## Abstract

Anticholinergic drugs can be used as a treatment for many diseases. However, anticholinergic drugs are also known for their cognition-related side effects. Recently, there has been an increasing number of reports indicating a positive association between exposure to anticholinergic drugs and Alzheimer's disease (AD). Our novel study provides evidence of interactions between two representative anticholinergic drugs [Chlorpheniramine (CPM), a common antihistamine, and Trazodone (TRD), an antidepressant] with neuroprotective amyloid-beta (Aβ) chaperone, lipocalin-type prostaglandin D synthase (L-PGDS) and the amyloid beta-peptide (1–40). Here, we demonstrate that CPM and TRD bind to L-PGDS with high affinity where chlorpheniramine exhibited higher inhibitory effects on L-PGDS as compared to Trazodone. We also show that the interactions between the drug molecules and Aβ(1–40) peptides result in a higher fibrillar content of Aβ(1–40) fibrils with altered fibril morphology. These altered fibrils possess higher cytotoxicity compared to Aβ(1–40) fibrils formed in the absence of the drugs. Overall, our data suggest a mechanistic link between exposure to anticholinergic drugs and increased risk of Alzheimer's disease *via* inhibition of the neuroprotective chaperone L-PGDS and direct modification of Aβ amyloid morphology and cytotoxicity.

## Introduction

Anticholinergic (AC) drugs can be defined as drugs directly targeting the muscarinic receptors in the cholinergic transmission pathway or drugs that contain anticholinergic properties (Coupland et al., 2019; Joung et al., 2019). This broad group of AC drugs can be used as a treatment for a wide range of diseases such as depression, an allergic reaction, and sleep (Joung et al., 2019). **Table 1** highlights the different kinds of commonly used drugs with AC effects. However, despite their therapeutic benefits in a variety of diseases, AC drugs are also known to have many side effects as they are capable of binding to other targets in the body (Lopez-Alvarez et al., 2019). Besides, most AC drugs are lipophilic which allows them to cross the blood-brain barrier (BBB) such as the first-generation antihistamine and anti-depressant to exert their effects in the brain (Chancellor et al., 2012). These properties of the AC drugs might have led to their adverse effects on the central nervous system which include cognitive decline and neurodegenerative diseases (Lopez-Alvarez et al., 2019). The AC effects of drugs can be categorized into two main events: the peripheral and the central events (Barkin and Stein, 1989). The peripheral events consist of angina, blurred vision, and urinary retention while the central events involve confusion, memory impairment and inability to concentrate. Among the different groups of the population that are exposed the AC drugs, the elderly patients are particularly sensitive to these AC side effects as many diseases that require AC medication are common to them (**Table 1**). Hence, the elderly patients are especially vulnerable to these potential AC side effects due to their frequent usages of the AC drugs.

**Table 1:** Some common medication with anticholinergic effects.

	Common medication
Depression	**Tricyclic Antidepressant** Amitriptyline (Elavil) Amoxapine (Asendin) Clomipramine (Anafranil) Desipramine (Norpramin) Doxepin (Sinequan) Imipramine (Tofranil) Nortriptyline (Pamelor) Protriptyline (Vivactil) Trimipramine (Surmontil) **Antidepressant (SSRI)** Fluoxetine (Prozac) Fluvoxamine (Luvox) Paroxetine (Paxil) Sertraline (Zoloft) Trazodone (Desyrel, Oleptro)
Anxiety	**Benzodiazepines** Alprazolam (Xanax) Chlordiazepoxide (Librium) Clonazepam (Klonopin) Clorazepate (Tranxene) Diazepam (Valium) Flurazepam (Dalmane, Dalmadorm) Lorazepam (Ativan) Oxazepam (Serax)
Allergies and cold	**Antihistamines** Azatadine (Optimine) Azelastine (Optivar) Brompheniramine (Dimetapp) Carbinoxamine (Ryvent, Palgic) Chlorpheniramine (Chlor-Trimeton) Clemastine (Tavist) Cyproheptadine (Periactin) Diphenhydramine (Benadryl) Hydroxyzine (Atrax, Vistaril) Triprolidine HCl (Actidil, Zymine)
Insomnia	**Benzodiazepines** Estazolam (ProSom) Quazepam (Doral) Temazepam (Restroil) Triazolam (Halcion) **Triazolopyridines** Nefazodone (Serzone) Trazodone (Desyrel)
Urge incontinence	**Antimuscarinics/Antipasmodics** Darifenacin (Enanblex) Fesoterodine (Toviaz) Flavoxate (Urispas) Oxybutynin (Ditropan) Solifenacin (Vesicare) Tolterodine (Detrol) Trospium (Sanctura)

Sources adapted from (Barkin and Stein, 1989; H.H. Publishing, 2015; Lopez-Alvarez et al., 2019).

Recently we determined the structure, dynamics, and catalytic mechanism of human lipocalin-type prostaglandin D synthase (L-PGDS) (Lim et al., 2013) and characterized its Aβ chaperone function (Kannaian et al., 2019) proposed by Kanekiyo et al. (Kanekiyo et al., 2007). We showed that L-PGDS inhibits nucleation and propagation of Aβ fibrils and is capable of disaggregation of pre-formed Aβ amyloids *in vitro* as well as in Alzheimer's disease (AD) brain extracts releasing proteins typically associated with amyloid plaques (Kannaian et al., 2019). L-PGDS is the second most abundant protein in human CSF (after albumin) with approximate concentrations of 26 mg/l (Reiber, 2001; Thompson, 2005). These pieces of evidence affirm the role of L-PGDS as a major neuroprotective Aβ chaperone and establish its importance in the AD pathogenesis. L-PGDS also exhibits a broad-range binding capacity for various small lipophilic ligands (Inui et al., 2003). Due to the high hydrophobicity of the protein, it has been proposed as a drug delivery vehicle to transport hydrophobic drugs from the gut to the brain (Fukuhara et al., 2012; Masatoshi Nakatsuji et al., 2015). Our 3D structure of human L-PGDS exhibits eight-stranded β-barrel with a deep calyx accommodating at least two lipophilic substrates, simultaneously (Lim et al., 2013; Kannaian et al., 2019). The monomeric Aβ peptides also bind at the entrance of the L-PGDS calyx with nanomolar affinity (Kanekiyo et al., 2007; Kannaian et al., 2019). We showed that L-PGDS might exert strong peroxidase activity when the first lipophilic ligand is iron-heme and that the produced reactive oxygen species might be contained within the enzyme by oxidizing the second ligand more peripherally or transiently bound to L-PGDS (Phillips et al., 2020). We posit that the strong ligand-binding ability of L-PGDS might facilitate the accumulation of AC drug molecules inside the calyx, their chemical modification (oxidation) and potentially interfere with the homeostasis of Aβ peptides associated with AD.

Several studies have shown that prolonged exposure to AC and AC related drugs such as antihistamine and antidepressants might significantly increase the risk of AD-associated dementia (Jessen et al., 2010; Coupland et al., 2019) (**Table 1**). However, the underlying molecular mechanisms of these associations remain unknown. In addition to the interference of L-PGDS chaperone activity, we also speculate that these hydrophobic AC drugs might be transported by L-PGDS to the Aβ peptide aggregation site. It has been reported that small lipophilic compounds are capable of binding to Aβ peptides and remodel the fibrils formed in the presence of these compounds (Du et al., 2015). Therefore, we propose that these AC drugs will exhibit off-target activity by directly interacting with the Aβ peptides and modify their morphology and cytotoxicity. The altered fibrils might result in higher cytotoxicity towards neurons thus exacerbating the risk of AD.

In our study, we have selected two most commonly used drugs from the anticholinergic family [e.g. Chlorpheniramine maleate (CPM) and Trazodone (TRD)]. CPM (**Figure S1B**) is a common over-the-counter (OTC), first-generation antihistamine drug. CPM tablets are used clinically for the treatment of allergic diseases and symptoms of a cold (Kirkegaard et al., 1983). Trazodone (TRD) (**Figure S1C**) is among the top 5 most prescribed antidepressant drugs in the United States (US) (Fuentes et al., 2018). Even though TRD was approved by the FDA as an antidepressant drug, it is one of the most widely prescribed off-label sleep aids in the US (Jaffer et al., 2017). The pharmacokinetics and pharmacodynamics properties of CPM and TRD are summarized in **Table 2**. We report the effects of these anticholinergic compounds on the neuroprotective function of L-PGDS and the direct interaction of these compounds with the Aβ(1–40) peptides resulting in altered amyloid morphology and increased cytotoxicity. In this study, tryptophan fluorescence quenching assays and Isothermal Titration Calorimetry (ITC) are carried out to characterize the binding of the selected compound to human L-PGDS. Thioflavin T (ThT) assays are performed to demonstrate inhibition of nucleation and fibril disaggregase functions of L-PGDS. Binding sites of the ligands to L-PGDS are mapped using NMR titrations. The potential alteration of the fibril morphology and biological activity induced by the drugs are studied using ThT assays, Transmission Electron Microscopy (TEM) and MTT cell toxicity assays.

**Table 2:** Pharmacokinetic and Pharmacodynamics properties of CPM and TRD.

	Chlorpheniramine (CPM)	Trazodone (TRD)
Mechanism of action	Competitive histamine H1 receptor antagonist	Serotonin type 2 (5-HT2) receptors, alpha1 (α1) adrenergic receptors antagonist and serotonin reuptake transporter inhibitor
Pro-drug	Chlorpheniramine Maleate	No
Dosing frequency	4 mg tablet, two times a day	50 to 150 mg per day
Bioavailability (%)	25 to 44 (Huang et al., 1982)	65 (Settimo and Taylor, 2018)
T_max_	2.08 h (Kotzan et al., 1982)	1.4 to 2.3 h
Half-life	13.9 to 43.3 h with multiple doses (Kirkegaard et al., 1983)	7.8 to 14.6 h
Metabolism	Mono- and didesmethyl-chlorpheniramine	m-chlorophenylpiperazine
Mode of excretion	20–26.5% of unchanged CPM excreted renally (Rumore, 1984)	70 to 75% excreted renally 21% excreted by the fecal route

NR, Not reported; Tmax, Time to maximum plasma concentration.

## Materials and Methods

Trazodone (TRD) (analytical grade) were purchased from Sigma Aldrich (Singapore). Chlorpheniramine, available as an OTC drug, was purchased from a local pharmacy store. Synthetic amyloid β (1–40) peptide was custom ordered from ChinaPeptides (China).

### Expression and Purification of Wild Type, Human Unlabeled L-PGDS

Glycerol stock of Rosetta 2 DE3, *E. coli* cells (Novagen) with pNIC-CH vectors were prepared *via* transformation. The rest of the protocol was followed from the paper (Lim et al., 2013). The concentrated protein was injected into the AKTA purifier Fast Performance Liquid Chromatography (FPLC) (GE Healthcare, USA), and further purified using Superdex 75 column in 50 mM sodium phosphate buffer (pH 7.0). Sodium dodecyl sulfate-polyacrylamide gel electrophoresis (SDS-PAGE) was run to check the purity of the different fractions obtained after the FPLC run.

### Expression and Purification of Wild Type, Human ^15^N Labeled L-PGDS

The glycerol stock of the *E. coli* cells used for the ^15^N labeled L-PGDS was the same as used for unlabeled L-PGDS. Starter culture was prepared in TB broth with kanamycin (50 mg/ml) and chloramphenicol (25 mg/ml) at 1:1,000 dilutions and inoculated with the glycerol stock containing the transformed cells. Expression, extraction, and purification of the ^15^N labeled L-PGDS was done in the same manner as the unlabeled L-PGDS.

### Tryptophan Fluorescence Quenching Assays

CPM and TRD were dissolved in dimethyl sulfoxide (DMSO) to give a 2.0 mM and a 20 mM stock solution respectively. Various concentrations of each compound were added to L-PGDS in 50 mM sodium phosphate (pH 7.0) buffer and the final concentration of the protein was adjusted to 2 µM. The maximum volume of DMSO in each 100 µl well was restricted to 2 µl (2%). The L-PGDS-compound-complex was formed by incubating L-PGDS with the respective drugs at 25 °C for 1 h. About 100 µl of each complex was added to the Corning Costar^®^ 96-well black, opaque, flat bottom plate. The intrinsic tryptophan fluorescence of L-PGDS was measured using Cytation 5 cell imaging multi-mode (Biotek Instrument Inc., USA) reader with λ_ex_ = 295 nm and λ_em_ = 340 nm. Duplicate measurements were performed for each concentration of the two AC drugs. Mass law equation was used to calculate the apparent dissociation constant (K_d_) values for the drug binding to L-PGDS using the following fitting equation (Eq. 1).

(1) $$F=\left(\left(\frac{\left(n*[Po])-[Lo]-Kd+\left(\left({\left(\left[Lo\right]+Kd-\left(n*\left[Po\right]\right)\right)}^{2}+{\left(4*Kd*n*\left[Po\right]\right)}^{\frac{1}{2}}\right)\right)*(Fo-F\min\right)}{2*n*[Po]}\right)+F\min\right)$$

### Isothermal Titration Calorimetry (ITC)

Calorimetric experiments were performed with Microcal ITC200 (Malvern, USA), in 50 mM sodium phosphate buffer (pH 7.0) at 25°C. L-PGDS (200 µM) in the injection syringe was reverse titrated into 2 µM CPM and 2 µM TRD in the cell. Titration experiments consisted of 19 injections spaced at 360 s intervals. The injection volume was 2 µl and the cell was continuously stirred at 750 rpm. The observed enthalpy changes (Δ*H*°) for binding and the dissociation constant (*K_d_*) were directly calculated and evaluated from integrated heats using one set of independent binding sites model available in MicroCal Origin 7.0 software (Kume et al., 2012).

### ThT Inhibition Assay

ThT dye (Sigma-Aldrich, Co) was dissolved in Milli Q water and filtered through a 0.2 µm syringe filter to obtain a stock concentration of 2.3 mM. The ThT dye was diluted to give a final working concentration of 200 µM. Aggregate-free Aβ(1–40) solution at a stock concentration of 250 µM was prepared by dissolving the synthetic Aβ(1–40) powder (ChinaPeptides Co., Ltd) in 1,1,1,3,3,3-Hexafluoro-2-propanol (HFIP). The HFIP in the mixture was left to evaporate overnight to obtain the dry powder. About 20 ul of 100 mM NaOH was added to the HFIP treated monomeric Aβ peptide powder to further dissolve the Aβ(1–40) and remove any pre-formed aggregates. After centrifuging at 14,000 rpm for 5 min, the supernatant was collected and 50 mM of sodium phosphate buffer was added to obtain a final working concentration of 50 µM monomeric solution of Aβ(1–40) peptide. The fluorescence intensity of ThT was measured using Cytation 5 cell imaging multi-mode (Biotek Instrument Inc., USA) reader with λ_ex_ = 430 nm and λ_em_ = 480 nm. The kinetics experiment was run for 72 h at 37 °C to monitor the formation of the Aβ(1–40) fibrils from the monomeric Aβ(1–40) peptide solution.

### ThT aggregation assay

The final concentration of monomeric Aβ(1–40) peptide was adjusted to 25 µM for the ThT aggregation assay with the drug molecules. This is to limit the volume of DMSO used in each well (<5%) as DMSO is known to interact with monomeric Aβ(1–40) peptide at high concentration.

### ThT Disaggregase Assay

Monomeric 200 µM, Aβ(1–40) peptide was grown at 37 °C shaking at 200 rpm for 72 h in an incubator to form the fibrils. For the control, 5 µM ThT dye was added to each well (100 ul) containing 10 µM preformed Aβ(1–40) fibrils. L-PGDS/drug complex was incubated for 30 min before adding to the wells. Duplicate measurements were performed for each condition on TECAN infinite M200 Pro microplate reader (Tecan Trading AG, Switzerland) with orbital shaking before each measurement. The kinetics experiment was run for 2 h at 37 °C to monitor the breakdown of the preformed Aβ(1–40) fibrils *via* L-PGDS and the L-PGDS/drug complexes. The percentage disaggregation of preformed Aβ(1–40) fibrils was calculated using the formula:

(2) $$P=\left[1-\left(\frac{Fa}{Fb}\right)\right]*100$$

where P is the percentage of disaggregation, *Fa* is the ThT fluorescence intensities of 10 µM preformed Aβ(1–40) fibrils and *Fb* is the ThT fluorescence intensities in the presence of L-PGDS or L-PGDS–CPM complex respectively.

### ^15^N Labeled Nuclear Magnetic Resonance (NMR) Titration

NMR titration experiments of L-PGDS with the CPM were acquired on Bruker Avance 700 MHz with triple resonance z-axis gradient cryoprobe at 298 K. All NMR samples contained 300 µM uniformly ^15^N-labeled L-PGDS (90%/10% D_2_O) in 50 mM sodium phosphate buffer (pH 7.0). Proton chemical shifts were referenced internally to 4, 4-dimethyl-4-silapentane-1-sulfonic acid (DSS) at 0.00 parts per million (ppm) with hetero nuclei referenced by relative gyromagnetic ratios. ^1^H-^15^N HSQC spectra of ^15^N-labeled L-PGDS (300 µM) was recorded in the absence and presence of drugs in a molar ratio of 1:1 and 1:4 (Protein : Ligand). These spectra were overlapped with the reference spectrum of L-PGDS alone to identify any chemical shift perturbations. Free induction decays were transformed and processed using Topspin (Bruker Biospin, USA). Assignment of the backbone nuclei of ^15^N labeled L-PGDS was carefully transferred from previously assigned spectra (Lim et al., 2013) and chemical shifts of the cross-peaks were analyzed using Computer-aided resonance assignment (CARA) (www.nmr.ch) (Keller, 2011). The chemical shift perturbation of the affected residues was calculated using the formula:

(3) $$\Delta \delta \;={\left\{\left[\Delta \delta {\left(H\right)}^{2}\right]+\;{\left[0.25*\;\Delta \delta \left(15N\right)\right]}^{2}\right\}}^{\frac{1}{2}}$$

and the resulting Δ*δ* was mapped onto the previously published L-PGDS crystal structure (PDB code: 4imn) to characterize the binding site of the drug—CPM onto the human WT L-PGDS.

### Transmission Electron Microscopy (TEM)

To visualize the direct interaction of CPM and TRD on the Aβ(1–40) fibrils formation, Aβ(1–40) monomer was incubated at 37°C for 72 h in 50 mM sodium phosphate buffer (pH 7.0). Aβ(1–40) fibrils samples treated with and without drugs were applied on copper–rhodium 400 mesh grids with 15 nm carbon coating (thickness) (prepared in-house) followed by negative staining with 2% uranyl acetate and then air-dried. The samples were then viewed under FEI T12. 120 kV Transmission electron microscope equipped with a 4K CCD camera (FEI) between 48,000× to 68,000× magnification under low dose conditions.

### MTT Metabolic Assay

The toxicity effects of all the amyloid fibrils were tested on the SH-SY5Y human neuroblastoma cell line using [3-(4,5-dimethylthiazol-2-yl)-2,5-diphenyltetrazolium bromide] (MTT) colorimetric assay. The cells were grown in 1:1 mix of Dulbecco's Modified Eagle Medium (DMEM) and Ham's F-12 growth media with 5% Fetal Bovine Serum (FBS) (Gibco) containing L-Glutamine and Phenol Red in 75 mm^3^ T-75 flasks at 37 °C in 5% carbon dioxide (CO_2_) environment till they achieved 70–90% confluency. After 24 h, approximately 2,500 cells were transferred into each reaction well of 96 well transparent tissue culture plate (Corning^®^ 96 Well TC-Treated Microplates). Once the cells were stabilized, 20 µl of samples were added in each well containing 100 µl culture media. Before the fibrils were added to the cells, they underwent a washing protocol to remove excess/unbound drug compounds as described previously (Palhano et al., 2013). The samples were added into the cell assay and incubated for 20 h to test their toxic effects. After incubation, 10 µl of the stock MTT reagent (5 mg/ml) was added to each well. After 4 h of conversion into the formazan product, DMSO was added to dissolve the purple crystals left in the dark for 1 h before the measurement of absorbance at 570 nm with 630 nm as a subtracted reference wavelength.

## Results

### Direct Interaction Between L-PGDS and Anticholinergic Drugs

**Figure 1:**
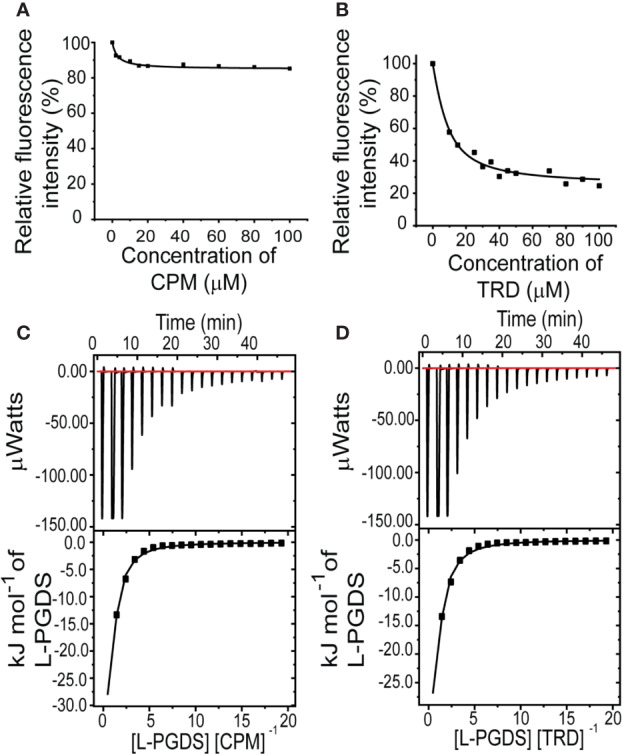
Interactions between L-PGDS and the two drugs—CPM and TRD. Tryptophan fluorescence quenching of L-PGDS monitored at 340 nm is plotted against the various concentrations of CPM **(A)**, TRD **(B)**. About 2 µM of L-PGDS in 50 mM sodium phosphate buffer pH 7.0 was used. Thermodynamic analysis of L-PGDS binding to CPM **(C)** and TRD **(D)** in 50 mM sodium phosphate buffer pH 7.0 by ITC. L-PGDS was reversely titrated to 2 µM of CPM and 2 µM of TRD. The top panes show thermograms and binding isotherms. The bottom panels show the change in heat obtained.

To assess the interaction between the anticholinergic (AC) drugs—CPM and TRD and L-PGDS, we performed the intrinsic tryptophan fluorescence quenching measurement on human wild-type L-PGDS in the presence of increasing concentrations of CPM and TRD. There are three tryptophan residues in human L-PGDS: Trp43 is located at the bottom of the L-PGDS cavity while Trp54 and Trp112 are located on the H2 helix and EF loop respectively (**Figure S1**) (Kume et al., 2012). Quenching of the intrinsic tryptophan fluorescence of L-PGDS was monitored as a function of the concentration of CPM and TRD (**Figures 1A, B**). The binding parameters obtained are summarized in **Table 3**. The difference in the intrinsic tryptophan quenching efficiency of the two drugs might be attributed to the different proximity of the ligand to the intrinsic tryptophan residues thus indicating possibly different binding sites of the two drug molecules.

**Table 3:** Binding affinities and thermodynamic parameters for interaction between L-PGDS with CPM and TRD.

Compound	Binding affinities and thermodynamic parameters					
	n	K_d_^a^ (µM)	ΔG (kJ mol^−1^)	ΔH (kJ mol^−1^)	-TΔS (kJ mol^−1^)	K_d_^b^ (µM)
CPM	3.0	5.7 ± 0.2	−42.9	−46.8 ± 0.04	3.9	4.3 ± 1.1
TRD	3.4	6.5 ± 0.2	−44.5	−48.6 ± 0.04	4.1	5.3 ± 1.0

n: Binding stoichiometry = number of molecules bound per protein molecule.

a: K_d_ obtained by ITC.

ΔG: Gibbs energy change.

ΔH: Enthalpy change.

ΔS: Entropy change.

b: K_d_ obtained by tryptophan fluorescence quenching of L-PGDS by binding the drugs.

To further confirm the binding and quantify the dissociation constant of the CPM/L-PGDS and TRD/L-PGDS complex we used the ITC assay. From the negative peaks of the titration curves obtained from ITC, binding of L-PGDS to all drugs was found to be an exothermic reaction, as also indicated by the favorable enthalpy changes, ΔH (**Figures 1C, D** upper panel). The integration of each peak area of the curve was plotted against the molar ratio ([L-PGDS] [DRUG]^−1^) (**Figures 1C, D** lower panel). The dissociation constant K_d,_ for the two drugs, were calculated by fitting the binding isotherms with the single set of identical sites binding model. The Kd obtained from ITC data are in good agreement with the K_d_ obtained from the tryptophan quenching data (**Table 3**).

### CPM Binds to L-PGDS and Inhibits Its Chaperone and Disaggregase Function

#### L-PGDS in Complex With CPM Exhibits Reduced Chaperone Functioning

**Figure 2:**
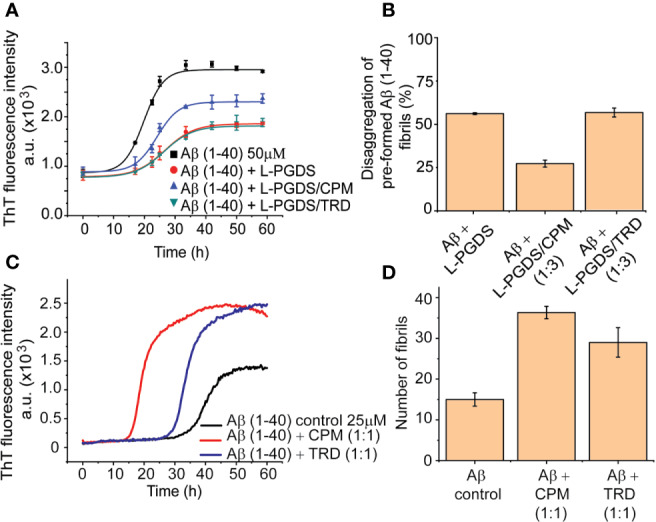
Inhibitory effects of L-PGDS and L-PGDS/drug complex on Aβ(1–40) peptide aggregation. **(A)** Representative time course of spontaneous Aβ(1–40) peptide aggregation in the absence (black) or presence of L-PGDS in 1:10 (protein:peptide) ratio (red) or presence of L-PGDS/CPM complex in ratio 1:3 (protein:drug) ratio (blue) or resence of L-PGDS/TRD complex in ratio 1:3 (protein:drug) ratio (green). **(B)** Disaggregation of preformed Aβ(1–40) fibrils by L-PGDS or L-PGDS/CPM complex in 1:3 (protein:drug) ratio and L-PGDS/TRD complex in 1:3 (protein:drug) ratio. Data shown are means ± SEM (n = 6). **(C)** Representative time course of spontaneous Aβ(1–40) peptide aggregation in the absence of compounds (black) or presence CPM in 1:1 ratio (red) or presence TRD in 1:1 ratio (blue). **(D)** Number of fibrils for each condition measured from the TEM images.

Our tryptophan quenching analysis and ITC results established the direct interaction between CPM and TRD with L-PGDS (**Table 3**). We have previously shown that human L-PGDS can interact with monomeric Aβ(1–40) peptide and prevent its aggregation (Kannaian et al., 2019; Phillips et al., 2020). Thus, to determine if the binding of the AC drug molecules affects the L-PGDS chaperone function we used the Thioflavin T (ThT) fluorescence assay to monitor the spontaneous aggregation of Aβ(1–40) peptide in the presence of L-PGDS/CPM and L-PGDS/TRD complex. Monomeric Aβ(1–40) peptide aggregation exhibits a characteristic sigmoidal curve indicative of amyloid aggregate formation *via* primary nucleation, fibril elongation, and secondary nucleation (Kannaian et al., 2019) (**Figure 2A**). The elongation phase of the control 50 μM monomeric Aβ(1–40) peptide starts at approximately 10 h after the lag phase reaching the steady phase at around 30 h which is typical for fibril formation (Xue et al., 2017). The presence of L-PGDS alone at 5 μM extends the elongation phase and decreases the final amount of Aβ(1-40) peptide aggregates to 40% when compared to the control (**Figure 2A**). However, in the presence of CPM in complex with L-PGDS (3:1, drug: protein molar ratio, binding stoichiometry obtained by ITC), L-PGDS exhibits lesser efficiency in reducing Aβ(1–40) aggregate formation as observed in **Figure 2A**. This could be indicative of partial overlap of the binding sites of the CPM drug molecule and monomeric Aβ peptide when interacting with L-PGDS. Thus, making L-PGDS/CPM complex less efficient as a chaperone. In contrast, the binding of TRD to L-PGDS did not result in any significant effect on L-PGDS chaperone activity (**Figure 2A**). We suspect this is due to TRD molecule binding to L-PGDS at a different site compared to Aβ peptide.

Another possibility for a less effective reduction of the ThT fluorescence intensity in the presence of the drug CPM could be due to direct interaction between the excess CPM molecule and the free monomeric Aβ(1–40) peptide itself. This interaction leads to an increase in the Aβ fibrils. To confirm this hypothesis, we performed the ThT fluorescence kinetic assay of the monomeric Aβ(1–40) peptides in the presence of CPM and TRD (**Figure 2C**). Indeed, both CPM and TRD, at a 1:1 molar stoichiometry, exhibited a sharp increase in the amount and rate of Aβ aggregate formation. A faster fibril formation with an overall higher fibrillar content as compared to Aβ(1–40) peptide control was observed (**Figure 2C**) in the presence of the two drug molecules. The elongation phase for 25 µM Aβ(1–40) monomeric peptide starts at ~15 h and ~25 h when incubated with CPM and TRD respectively, as compared to ~30 h for the 25 μM Aβ(1–40) control. Both CPM and TRD appeared to have shortened the lag phase while significantly increasing the total fibril content (**Figure 2D**). The increase in the fibril content was also confirmed by Transmission Electron Image analysis (**Figures S4A–C**). The electron micrographs obtained from the TEM were analyzed using Image J software to quantify the number of both modulated and control fibrils of Aβ(1–40, Kannaian et al., 2019).

#### Binding to CPM Lowers the Disaggresase Activity of L-PGDS

Our previous data have demonstrated the unique ability of human WT L-PGDS to break down preformed Aβ(1–40) fibrils (Kannaian et al., 2019). To further examine the effect of CPM binding to L-PGDS on its disaggregase function, we monitored disaggregation of the pre-formed Aβ(1–40) peptide fibrils in the presence of L-PGDS and (1:3) L-PGDS/CPM complex. L-PGDS alone efficiently breaks down up to 50% of the preformed Aβ(1–40) peptide fibrils resulting in a decreased ThT fluorescence intensity. Whereas, the addition of L-PGDS/CPM (1:3) complex to the pre-formed Aβ (1–40) peptide fibrils only breaks down up to 30% of the Aβ(1–40) peptide fibrils (**Figure 2B**). As expected from the binding site of TRD in L-PGDS, L-PGDS/TRD (1:3) complex did not show any significant inhibitory effect on the disaggregases activity of L-PGDS (**Figure 2B**). The possibility of the presence of excess CPM molecules interfere with the effective breakdown of the preformed Aβ fibrils by L-PGDS can be ruled out because TRD which also showed to be interacting with Aβ fibrils in the previous assay did not show any significant effects on the breakdown of fibrils. Thus, indicating that the presence of excess drug molecules has a negligible effect on the effective breakdown of fibrils.

#### CPM Binding Site Overlaps With Aβ(1–40) Peptide-Binding Site on L-PGDS

**Figure 3:**
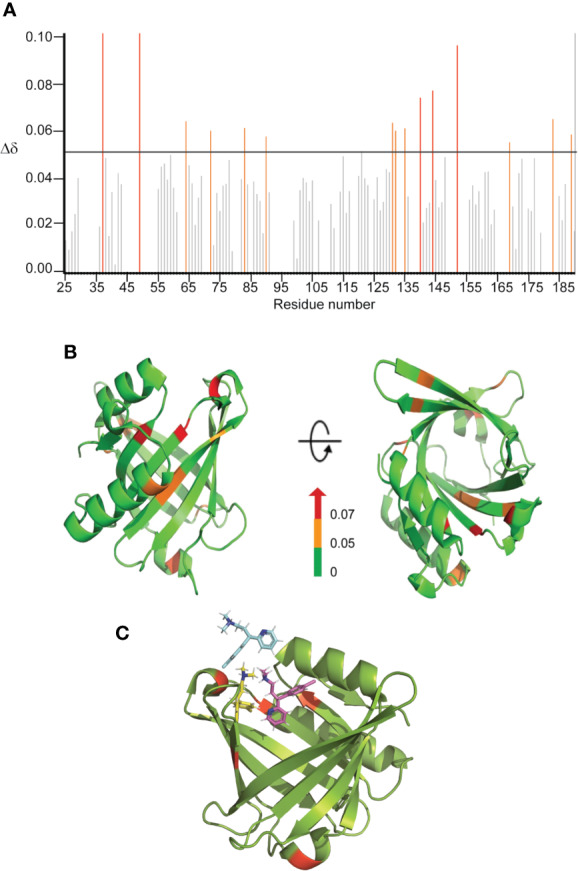
Binding sites characterization of CPM on L-PGDS, as determined by NMR titrations. **(A)** The chemical shift perturbations (Δδ) of backbone and amide groups of residues in L-PGDS induced by binding to CPM at a molar ratio of 1:4 (protein:drug). **(B)** Mapping of chemical shift perturbations (Δδ) on L-PGDS crystal structure (PDB code: 4IMN) in the presence of CPM (1:4) (protein:ligand). Red and orange show Δδ >0.07 and 0.05 < Δδ < 0.07, respectively. In the panels, left and right images are the front and top views of L-PGDS (PDB code: 4IMN) in solution, respectively. **(C)** Model of three CPM molecules (blue, yellow and magenta) docking onto L-PGDS (green) obtained from online protein-ligand docking platform, HADDOCK (van Zundert et al., 2016).

Since CPM displayed a more significant effect on both the chaperone and disaggregase activity of L-PGDS, characterization of the CPM binding site to L-PGDS was of greater interest to us. To achieve this, we acquired NMR spectra—2D ^1^H-^15^N HSQC of ^15^N labeled L-PGDS in the absence (apo) and presence of increasing concentrations of CPM (**Figure S2**). The assignment for the reference spectrum was carefully transferred from previously published data recorded in the same sample conditions (Lim et al., 2013). The residues with average chemical shift perturbations higher than a preset threshold of 0.05 ppm (**Figure 3A**) were mapped onto the crystal structure of L-PGDS (PDB) code: 4IMN) (Lim et al., 2013) to define the interaction site of CPM with L-PGDS. Residues M64, A72, F83, E90, L131, Y132, G135, A169, T183 and E189 showed moderate chemical shift perturbations (0.05 < Δδ < 0.07) and are highlighted in orange. Residues D37, A49, G140, R144, and T152 showed large chemical shift perturbations (Δδ > 0.07) and are highlighted in red. Overall, CPM binding to L-PGDS results in significant chemical shift perturbations mainly originating from the residues located at the entrance of the β-barrel cavity of L-PGDS (**Figure 3B**). The tryptophan fluorescence quenching results also point to similar binding sites for CPM positioned away from W43.

The residues D37, A49, G140, R144 and T152 identified from our NMR titration analysis were used to guide the docking of CPM molecules into the crystal structure of L-PGDS (PDB: 4IMN) using HADDOCK, an online protein-ligand docking platform (van Zundert et al., 2016). Our docking model demonstrates CPM molecules binding near the β-calyx entrance of L-PGDS as shown in **Figure 3C**. The entrance of the β-calyx of L-PGDS can accommodate up to three CPM molecules, thus supporting the stoichiometric binding ratio estimated from our ITC measurements. Besides, the distance between the CPM molecule and Trp43 in L-PGDS was calculated based on our docking models (**Figures S3A, B**). This information helps to support out previous Tryptophan quenching data where CPM showed a lower quenching efficiency as compared to TRD.

#### The Binding of the AC Drug Molecules Remodels the Aβ(1–40) Peptide Fibril Morphology and Increases its Cytotoxicity

It has been reported that small compounds such as brazilin and EGCG are capable of remodeling the amyloid fibrils and reducing the cytotoxicity of the fibril (Bieschke et al., 2010; Du et al., 2015). Since we have established that the drug molecules interact with the Aβ peptides, CPM and TRD might also remodel the mature amyloid fibrils and affect the resultant amyloid cytotoxicity. Hence, we went on to investigate the direct interaction between the selected drugs and the Aβ(1–40) and analyze the effect of the drugs on the amyloid cytotoxicity of Aβ(1–40).

**Figure 4:**
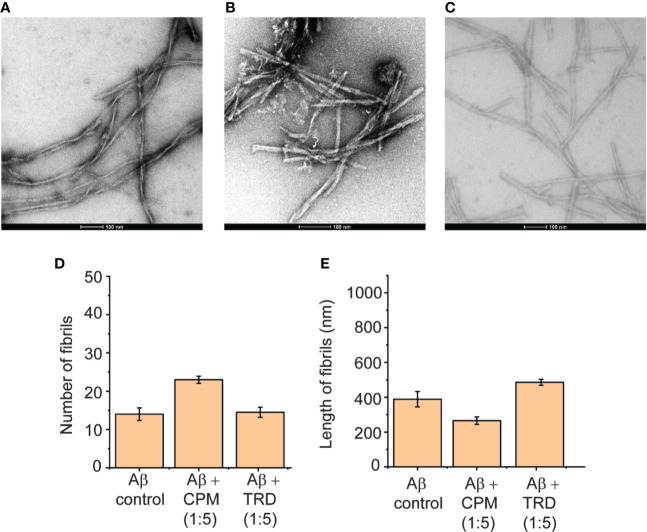
Direct interaction between CPM and TRD with Aβ(1–40) peptides in a 1:5 ratio. **(A)** TEM image of Aβ control grown for 60 h at 37°C under continuous shaking. **(B)** TEM image of 50 µM Aβ control grown in the presence of 250 µM CPM (1:5 ratio) for 60 h at 37°C under continuous shaking. **(C)** TEM image of Aβ control grown in the presence of 250 µM TRD (1:5 ratio) for 60 h at 37°C under continuous shaking. **(D)** Number of fibrils for each condition measured from the TEM images. **(E)** Length of fibrils for each condition measured from the TEM images.

Upon further increase in the concentration of CPM and TRD to 1:5 (Aβ: drug), modulation of the Aβ(1–40) peptide fibrils were observed. For CPM, in addition to the increase in the total fibril content, the fibrils also became shorter and thicker in terms of width (**Figures 4B, D, E**) when compared to the control fibrils. The fibrils grown in the presence of TRD did not show any significant visible differences in the number and morphology when compared to the control (**Figures 4C–E**). Since it has been shown that these compounds directly interacted with the fibrils with CPM capable of changing the morphology of the resultant fibrils, we speculate that the remodeled fibrils may show different biological activities of the resulting Aβ fibrils as it was demonstrated in the subsequent cell viability assays.

**Figure 5:**
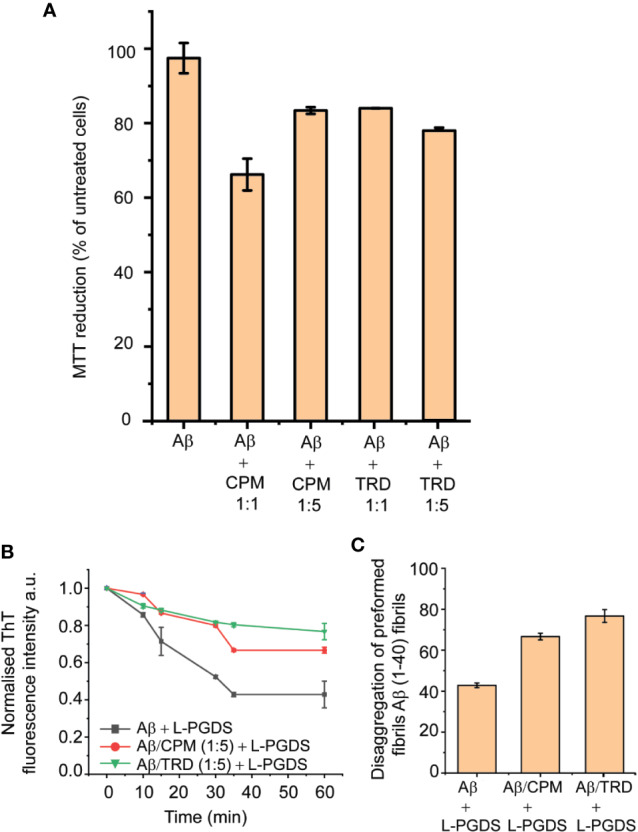
Toxicity and Biological activities of modulated fibrils. **(A)** Various effect on the metabolic activity of SH-SY5Y cells after addition of modulated fibrils. Fibrils were grown in the presence of compounds for 72 h at 37°C and added into the cell culture media. MTT reduction was normalized to control cells. Values represented means ± SEM. **(B)** Representative time course of disaggregation of fibrils grown in different conditions. Control Aβ fibrils with the addition of 5 µM of L-PGDS (black), Aβ fibrils grown in 250 µM of CPM with the addition of 5 µM of L-PGDS (red), fibrils grown in 250 µM of TRD with the addition of 5 µM of L-PGDS (green). **(C)** Disaggregation of preformed Aβ(1–40) fibrils by L-PGDS, Aβ fibrils grown in 250 µM of CPM (1:5 peptide:drug ratio) and Aβ fibrils grown in 250 µM of TRD (1:5 peptide:drug ratio). Data shown are means ± SEM (n = 6).

The 3-(4,5-dimethylthiazol-2-yl)-2,5-diphenyl tetrazolium bromide (MTT) metabolic assay was employed to investigate the cytotoxicity of the Aβ(1–40) fibrils grown in the presence of the AC drugs—CPM and TRD in neuron-like SH-SY5Y human neuroblastoma cells. The Aβ(1–40) fibril control did not exhibit any significant effect on the viability of the SH-SY5Y cells as also shown previously by Krishtal et al. (2017). In contrast, exposure to Aβ(1–40) fibrils grown in the presence of CPM in a molar ratio of 1:1, reduced the survival of SH-SY5Y cells to 66.19% of the control. Interestingly, the Aβ(1–40) fibrils grown in the presence of higher CPM concentration (molar ratio of 1:5 for LPGDS: CPM) only induced a slight decrease in cell viability (83.39% of the control). For Aβ(1–40) fibrils grown in the presence of TRD in a ratio of 1:1 and 1:5, drop-in cell viability to ~83% of the control was observed (**Figure 5A**). Overall, the MTT assays indicated that the altered Aβ(1–40) fibrils exhibit various toxicity levels when compared to the untreated Aβ(1–40) fibril control most likely due to the different morphology of the affected fibrils.

The remodeled Aβ(1–40) fibrils also exhibited different effects on the disaggregase function of L-PGDS. From the ThT result, it can be seen that the fibrils formed in the presence of both AC drugs display different activity as compared to the Aβ(1–40) control fibrils, where they interfere with the disaggregation efficiency of L-PGDS as shown in **Figures 5B, C**. The fibrils grown in the presence of TRD showed the greatest resistance against the disaggregase action by L-PGDS where ~76% of the fibrils remain intact after incubation with L-PGDS. Fibrils were grown in the presence of CPM also showed reduced susceptibility (~66%) to L-PGDS disaggregase activity albeit to a lesser extent (**Figure 5C**).

## Discussion

Using the unintended interaction of AC drugs with L-PGDS and Aβ peptide, we found the possible link between prolonged exposure of AC drugs with increased risk in AD. In this study, we selected two representatives AC drug compounds and explored their ability to bind to L-PGDS, interfere with its amyloid nucleation and propagation inhibition as well as its amyloid disaggregase functions. Also, we show that at elevated concentrations, which are still within the physiological range found in the brain tissues, these drugs are capable of remodeling the resulting Aβ fibrils typically increasing their resistance to disaggregation and cytotoxicity. Reported here is our first study pointing to the direct mechanistic link between exposure to AC drugs and change in the homeostasis of Aβ peptides typically associated with AD.

Interestingly, although both drugs bind to L-PGDS with similar affinities, CPM is the only compound that interferes with the interaction between the Aβ(1–40) peptide and L-PGDS (**Figure 2A** and **Figure S2**) most likely inhibiting the binding of Aβ(1–40) monomers to L-PGDS. While interacting with Aβ (1–40), the chemical shifts of residues D37 and R144 of L-PGDS were significantly perturbed and the resonances of A49 and G140 were broadened beyond detection (Kannaian et al., 2019). The MD simulation model developed in our previous study showed that Aβ(1-40) peptide interacts with L-PGDS at the entrance of the L-PGDS calyx (Kannaian et al., 2019). In this study, WT L-PGDS in complex with CPM showed large chemical shift perturbations for similar residues (**Figure 3A**) and our docking model revealed CPM molecule binds to the entrance of the L-PGDS calyx (**Figure 3C**) similar to Aβ(1–40). Thus, we propose that CPM exerts its inhibitory effect on the chaperone activity of WT L-PGDS by either partially or fully occupying the Aβ(1–40) peptide-binding site on L-PGDS. It is also possible that CPM binding to different regions of L-PGDS results in a conformational change within L-PGDS (as shown in the large chemical shifts observed in the bottom of the calyx (**Figure 3B**) leading to allosteric inhibition of L-PGDS's ability to bind Aβ(1–40) monomers, oligomers or even fibrils. Further studies necessitate the need to investigate the detailed mechanism that involves the specific inhibitory effect of CPM on the L-PGDS function. This might provide further insights into how other similar AC drugs interfere with the chaperone network within AD. The compound specificity observed in L-PGDS inhibition could also provide reasons as to why certain AC drugs showed a stronger association with AD than the rest.

Both CPM and TRD were found to directly interact with the Aβ peptide as demonstrated by the sharp increase in ThT fluorescence assays (**Figure 2C**). The resulting fibrils have higher fibrillar content and longer fibrils as compared to the control (**Figures 2C, D**). We propose that at a lower concentration of the selected drugs, these drugs interact with the Aβ(1–40) peptide and affect the nucleation mechanism of the Aβ peptide. The shortening of the lag phase of the ThT curve in the presence of these drugs (**Figure 2C**) supports our claim that the compounds were accelerating the nucleation process of Aβ peptides as nucleation normally occurs at the lag phase of the aggregation curve for Aβ fibrils (Kumar and Walter, 2011). As Aβ fibrils grow *via* the addition of monomers at fibrils' end (Cohen et al., 2013), it is likely that these drugs interact with the short axis of the fibril and cause the elongation of the fibrils. Hence, the fibrils grown in the presence of the drugs had higher fibril content and the length of the fibrils was significantly longer. With regards to the change in morphology of the fibrils at higher concentrations of the drugs (1:5; Aβ(1–40) peptide: drugs), we propose that a large number of drugs present at such concentrations is sufficient to interact with the long axis of the fibrils (long side interactions). This interaction between the fibrils and drugs would affect the fibril packing resulting in the change in morphology of the fibrils (Close et al., 2018) as observed in TEM images (**Figures 4A–C**).

From the results of the MTT (**Figure 5A**) and ThT assays (**Figures 5B, C**), we conclude that different remodeling effects of fibrils caused by the interaction of the drugs can exhibit different toxicities and biological activities. We suggest that when the fibrils are remodeled by the drugs, different sets of amino acids would be buried in the core of the fibrils. Hence, causing an alteration of the interaction sites of the modulated fibrils, leading to the fibrils displaying different toxicity and biological activity as compared to the unmodulated fibrils. This hypothesis is further supported by Petkova et al. where they also suggested that the reason for different morphology leading to different biological activities could be due to the exposure of different amino acid side chains on the fibril surface (Petkova et al., 2005). It is, therefore, worth exploring with future studies on the detailed structure of the modulated fibril to unravel the relationship between fibril morphology and its respective toxicity and biological activity. Furthermore, it has been reported that the toxicity of Aβ fibrils could be related to the process in which the fibrils are grown during the initial nucleation process (Jan et al., 2011). Since we have shown in our experiment that the drugs most likely interact with the Aβ monomers during the fibrillation process (**Figure 2C**), the molecular mechanism on how these drugs interact and alter the structure of monomers and fibrils might also provide valuable insights for the different toxicity observed in this study. Regarding the possible reversibility of the interaction between the drug molecules and Aβ peptide, our studies displayed a greater resistance of the altered fibrils towards the disaggregase activity of L-PGDS. Thus suggesting an irreversible drug–Aβ (1–40) peptide interaction that is probably retained even after the discontinuation of the AC drugs.

Notably, most AC drugs association studies with AD mainly focus on prescribed AC drugs (Gray et al., 2015; Joung et al., 2019). However, in our study, we observed that out of the two AC drugs, CPM which is available as an OTC drug seems to have a markedly stronger inhibitory effect on L-PGDS and result in higher toxicity in the modulated fibrils. Hence, our study also highlights the importance to take the biological effects of non-prescribed/OTC AC drugs into consideration when measuring the impact of AC drugs on AD patients. It is important to pay careful attention to the potential AC side effects such as peripheral and central events of AC drugs and increased risk in AD before using OTC or prescribed AC drugs as a treatment for diseases, especially to elderly patients.

## Conclusion

Together, our studies show that the unintended binding and biological properties of AC drugs could be the missing link between exposure to AC drugs and increased risk in AD. We discovered that AC drugs such as CPM and TRD bind to lipophilic carriers such as L-PGDS with relatively high affinities and disrupt its neuroprotective function important for the Aβ homeostasis. We also showed that these drugs are capable of directly interacting with the amyloid fibrils and remodeling their morphology. This change in morphology resulted in varying toxicity of the modulated fibrils and their respective resistance towards the disaggregation of L-PGDS. Our study mainly focuses on the *in vitro* effects of the AC drugs on neuroprotective chaperone and Aβ. Hence, our results might not be easily translated into the *in vivo* systems due to inherent complexity of the organ systems. Future *in vivo* studies such as animal models, may provide a more detailed mechanism to describe the link between AC drugs and increased risk in AD. This study aims to trigger further interest in understanding other mechanisms that might link other drug compounds with increased AD risk. The morphological and toxicity differences of the modulated fibrils induced by AC drugs may serve as a useful model for investigating the relationship between amyloid fibril structure and the resulting biological activities observed in our study.

## Data Availability Statement

The raw data supporting the conclusions of this article will be made available by the authors, without undue reservation, to any qualified researcher.

## Author Contributions

KL: Writing—protein sample preparation and performed all biophysical experiments and analyzed all data. MP: Conceived and designed the experiments, performed NMR acquisition, and TEM data acquisition. KP: Conceptualization, Writing—review and editing, Supervision, project administration, and funding acquisition. All authors contributed to the article and approved the submitted version.

## Conflict of Interest

The authors declare that the research was conducted in the absence of any commercial or financial relationships that could be construed as a potential conflict of interest.
