# Supplementary material for: Anticholinergic Drugs Interact With Neuroprotective Chaperone L-PGDS and Modulate Cytotoxicity of Aβ Amyloids
Source: Front Pharmacol. 2020 Jun 11;11:862. doi: 10.3389/fphar.2020.00862 (PMC7300299; doi:10.3389/fphar.2020.00862)
Supplement: Supplementary file 1 [file Image_1.pdf]

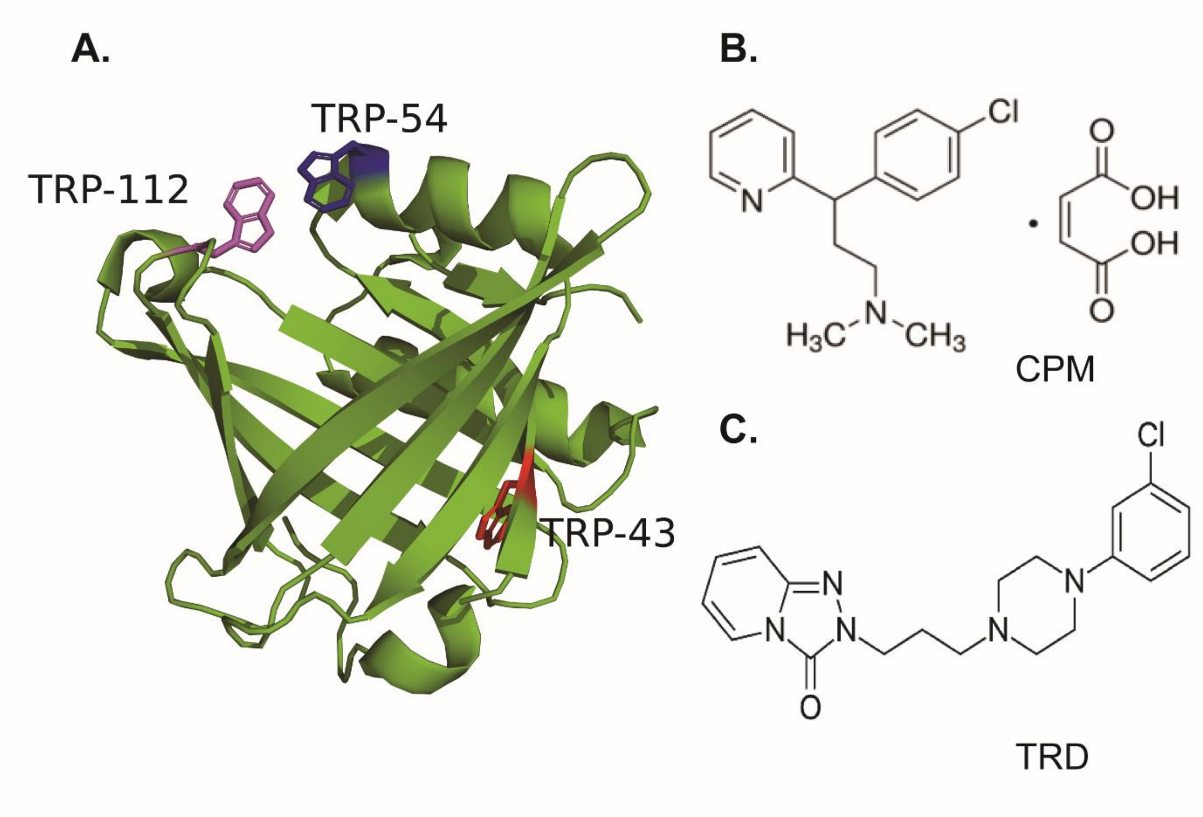


Figure S1: Structure of L-PGDS and the anticholinergic drug compounds CPM and TRD. (A) Structure of human WT L-PGDS (PDB ID: 4IMN) [1] the three different tryptophan residues of L-PGDS: Trp43, Trp54 and Trp112 are shown in red, blue and magenta respectively. The chemical structures of CPM (B) and TRD (C).

[1] S.M. Lim, D. Chen, H. Teo, A. Roos, A.E. Jansson, T. Nyman, L. Tresaugues, K. Pervushin, and P. Nordlund, Structural and dynamic insights into substrate binding and catalysis of human lipocalin prostaglandin D synthase. Journal of lipid research 54 (2013) 1630-43.
