# Supplementary material for: Anticholinergic Drugs Interact With Neuroprotective Chaperone L-PGDS and Modulate Cytotoxicity of Aβ Amyloids
Source: Front Pharmacol. 2020 Jun 11;11:862. doi: 10.3389/fphar.2020.00862 (PMC7300299; doi:10.3389/fphar.2020.00862)
Supplement: Supplementary file 3 [file Image_3.pdf]

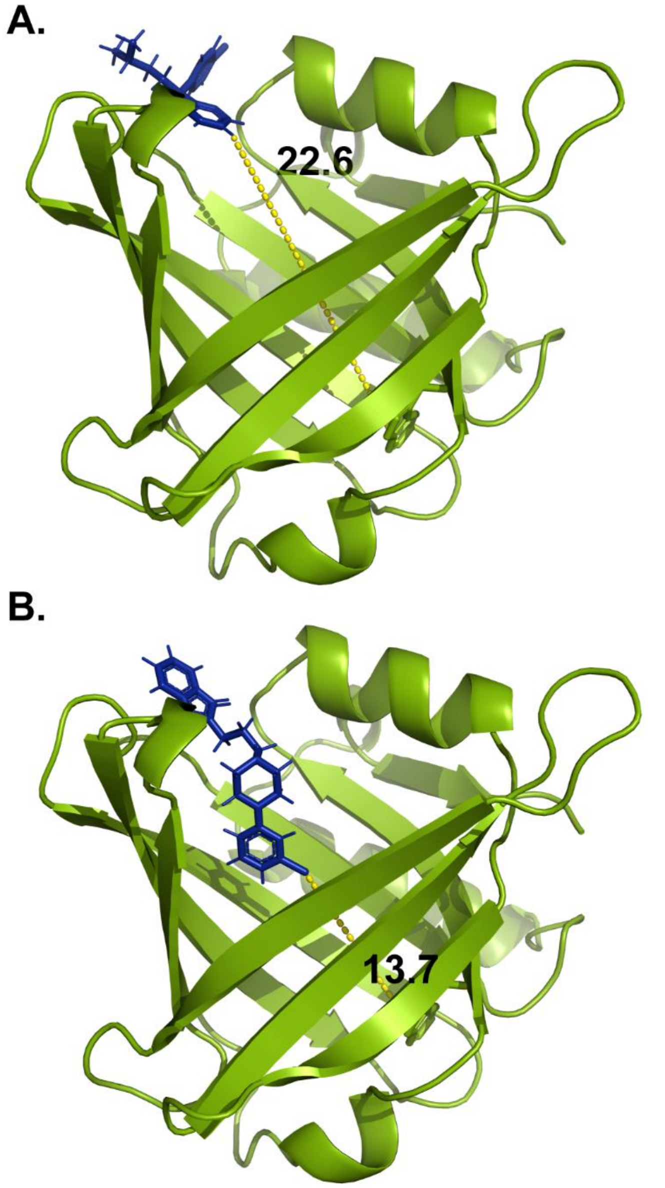

Figure S3: Model of the L-PGDS/drug complex obtained from SwissDock [1] using the crystal structure of L-PGDS (PDB ID: 4IMN) [2] (A) L-PGDS/CPM complex with the distance between CPM and the Trp43 residue of L-PGDS highlighted in yellow. (B) L-PGDS/TRD complex with the distance between TRD and the Trp43 residue of L-PGDS highlighted.

[1] G.C.P. van Zundert, J. Rodrigues, M. Trellet, C. Schmitz, P.L. Kastritis, E. Karaca, A.S.J. Melquiond, M. van Dijk, S.J. de Vries, and A. Bonvin, The HADDOCK2.2 Web Server: User-Friendly Integrative Modeling of Biomolecular Complexes. J Mol Biol 428 (2016) 720-725.

[2] S.M. Lim, D. Chen, H. Teo, A. Roos, A.E. Jansson, T. Nyman, L. Tresaugues, K. Pervushin, and P. Nordlund, Structural and dynamic insights into substrate binding and catalysis of human lipocalin prostaglandin D synthase. Journal of lipid research 54 (2013) 1630-43.
