# Supplementary material for: Anticholinergic Drugs Interact With Neuroprotective Chaperone L-PGDS and Modulate Cytotoxicity of Aβ Amyloids
Source: Front Pharmacol. 2020 Jun 11;11:862. doi: 10.3389/fphar.2020.00862 (PMC7300299; doi:10.3389/fphar.2020.00862)
Supplement: Supplementary file 4 [file Image_4.pdf]

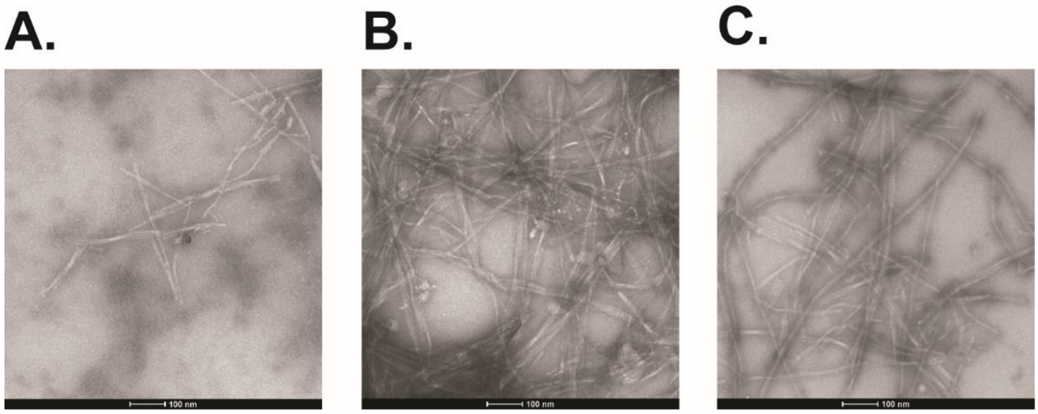

Figure S4: Direct interaction of drugs between CPM and TRD with Aβ(1-40) peptides in a 1:1 ratio. (A) TEM image of Aβ(1-40) peptide control grown for 60 h at 37°C under continuous shaking (B) TEM image of Aβ(1-40) control grown in the presence of CPM (1:1 ratio) for 60 h at 37°C under continuous shaking. (C) TEM image of Aβ(1-40) control grown in the presence of TRD (1:1 ratio) for 60 h at 37°C under continuous shaking.
