# Supplementary material for: Anticholinergic Drugs Interact With Neuroprotective Chaperone L-PGDS and Modulate Cytotoxicity of Aβ Amyloids
Source: Front Pharmacol. 2020 Jun 11;11:862. doi: 10.3389/fphar.2020.00862 (PMC7300299; doi:10.3389/fphar.2020.00862)
Supplement: Supplementary file 2 [file Image_2.pdf]

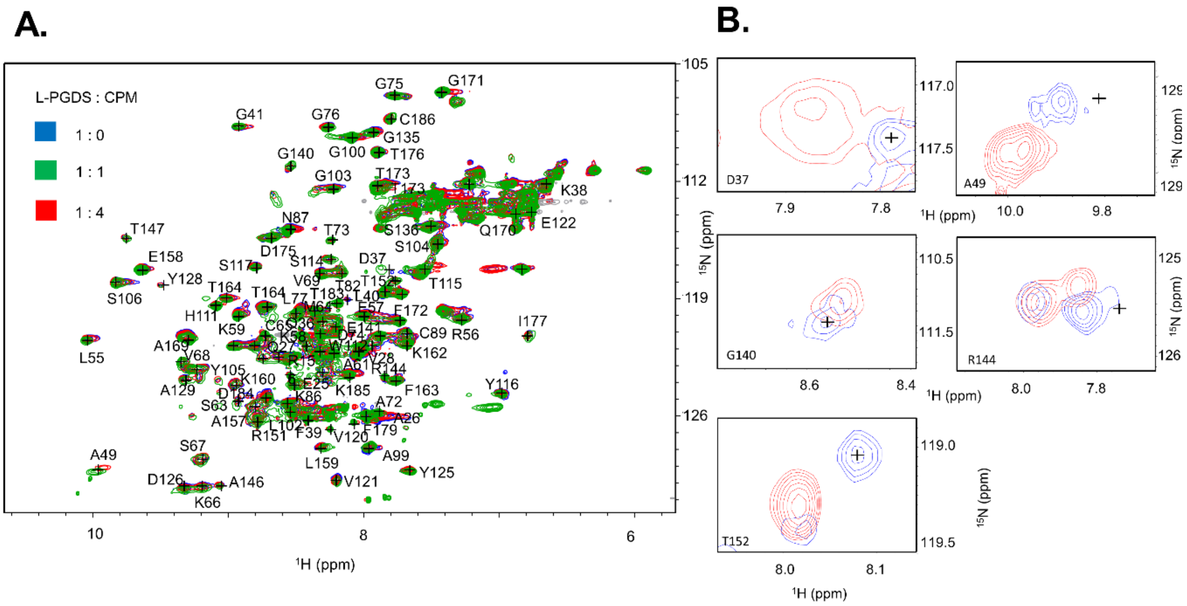

Figure S2: NMR titration of ^{15}N-labeled L-PGDS with unlabelled CPM. (A) Superposition of 2D-HSQC spectra of 15N-labeled L-PGDS alone (blue), upon addition of CPM (blue) at a molar ratio of 1:1 and upon addition of CPM (red) at a molar ratio of 1:4 (B) Resonances undergoing significant chemical shift perturbations in the 1:4 titration step have been highlighted.
